# Effects of different inspiratory muscle warm-up loads on mechanical, physiological and muscle oxygenation responses during high-intensity running and recovery

**DOI:** 10.1038/s41598-022-14616-w

**Published:** 2022-07-02

**Authors:** Anita B. Marostegan, Claudio A. Gobatto, Felipe M. Rasteiro, Charlini S. Hartz, Marlene A. Moreno, Fúlvia B. Manchado-Gobatto

**Affiliations:** 1 Laboratory of Applied Sport Physiology, School of Applied Sciences, University of Campinas, Limeira, São Paulo Brazil; 2 Postgraduate Program in Human Movement Sciences, Methodist University of Piracicaba, Piracicaba, Sao Paulo Brazil

**Keywords:** Infrared spectroscopy, Physiology

## Abstract

Inspiratory muscle warm-up (IMW) has been used as a resource to enhance exercises and sports performance. However, there is a lack of studies in the literature addressing the effects of different IMW loads (especially in combination with a shorter and applicable protocol) on high-intensity running and recovery phase. Thus, this study aimed to investigate the effects of three different IMW loads using a shorter protocol on mechanical, physiological and muscle oxygenation responses during and after high-intensity running exercise. Sixteen physically active men, randomly performed four trials 30 s all-out run, preceded by the shorter IMW protocol (2 × 15 breaths with a 1-min rest interval between sets, accomplished 2 min before the 30 s all-out run). Here, three IMW load conditions were used: 15%, 40%, and 60% of maximal inspiratory pressure (MIP), plus a control session (CON) without the IMW. The force, velocity and running power were measured (1000 Hz). Two near-infrared spectroscopy (NIRS) devices measured (10 Hz) the muscle’s oxygenation responses in biceps brachii (BB) and vastus lateralis (VL). Additionally, heart rate (HR) and blood lactate ([Lac]) were also monitored. IMW loads applied with a shorter protocol promoted a significant increase in mean and minimum running power as well as in peak and minimum force compared to CON. In addition, specific IMW loads led to higher values of peak power, mean velocity (60% of MIP) and mean force (40 and 60% of MIP) in relation to CON. Physiological responses (HR and muscles oxygenation) were not modified by any IMW during exercise, as well as HR and [Lac] in the recovery phase. On the other hand, 40% of MIP presented a higher tissue saturation index (TSI) for BB during recovery phase. In conclusion, the use of different loads of IMW may improve the performance of a physically active individual in a 30 s all-out run, as verified by the increased peak, mean and minimum mechanical values, but not in performance assessed second by second. In addition, 40% of the MIP improves TSI of the BB during the recovery phase, which can indicate greater availability of O_2_ for lactate clearance.

## Introduction

High-intensity exercise such as tethered sprints is often applied in training programs^1–3^. Considering the significance of the running power in sports and training programs, investigations have been conducted to improve the consistency of this parameter by making it measurable using a tethered system capable of monitoring the force, the velocity, and consequently the running power^2,4–6^. Furthermore, by applying a high-intensity exercise test, especially with duration around 30 s (i.e., 30 s all-out run), it is possible to assess the peak, mean and minimum power, as well as the fatigue index^7–9^, which contribute to training programs applied to athletes and active participants. The 30 s all-out run is characterized by a predominantly anaerobic stimulus that increases the physiological responses, such as heart rate and blood lactate production^7,8,10^, by locomotor muscles (more active muscles). Despite the significance of the anaerobic metabolism, the aerobic metabolism also plays an important role during high-intensity exercise, as observed in the Wingate test^11–13^ and the 30 s all-out tethered run^7^. In addition, the recovery process is essential to reestablish the energy stores, with significant participation of the aerobic metabolism during and after high-intensity exercise ^10,14^, acting as an ally in the performance of subsequent intense efforts^15^. Recently, our group observed that during an all-out 30 s running test the biceps brachii (less active muscle) showed increased deoxygenation and a quick adjustment of post-effort oxygen levels compared to the vastus lateralis^10^. These findings reinforce the tissue-dependent response, evidencing that the organism adapts to a stressful stimulus in an integrative way.

Considering the integrative biology during physical effort^16^ in high-intensity exercise, it is known that the oxygenation is directed to areas with higher demand, promoting vasoconstriction in less active muscles and vasodilation in more active muscles. In this sense, the accumulation of metabolites (e.g., blood lactate and H + ions) in the fatigued inspiratory muscles (IM) can initiate the inspiratory muscle metaboreflex, which triggers sympathetic nerve activity that promotes adrenergic vasoconstriction, provoking competition with locomotor muscles for oxygenation^17–20^, and consequently impairing the exercise performance. In line with this, it is reported that IM plays a significant role during exercise and recovery^19–22^. Therefore, some studies have focused on IM training, aiming at reducing the IM fatigue in order to maximize performance and oxygenation redistributions^23–25^. Moreover, the warm-up or pre-activation of inspiratory muscles (IMW) using a portable device has been suggested to prepare this specific musculature before exercise, and consequently improve the physiological responses during effort^26–28^, as well as sports performance^29–32^. Positive effects of IMW were observed during high-intensity, short-duration exercises, with increased power in the Wingate test^33,34^. Furthermore, better oxygenation distributions in more active muscles^35^ and decreased lactate accumulation in athletes after intermittent running^36^ were also observed. However, the available literature lacks studies on the effects of IMW on more and less active muscles, which are essential for oxygen uptake and metabolite removal, respectively^10,37^.

Different IMW protocols (i.e., number of sets, repetitions and time between sets) and loads (characterized by airflow restriction in the inspiration phase) ranging from 5 to 80% of maximal inspiratory pressure (MIP) have been proposed^29,31,32,38–41^. Nonetheless, most studies that applied this strategy used efforts based on individual MIP, generally composed of 2 sets of 30 repetitions at 40% of MIP. This protocol has been suggested to improve exercise performance through the preparation of the inspiratory muscles, to minimize the effects of inspiratory muscle metaboreflex, reducing fatigue and improving oxygen delivery between the locomotor and respiratory muscles^29,31,34,40^. The application of higher inspiratory loads such as 60–80% of MIP^38,39^ to improve subsequent results is still controversial and lower loads such as 5–15% of MIP are commonly applied as a placebo condition^27,31,35,36,39^. Concerning the number of repetitions, recent investigations have suggested a shorter IMW protocol consisting of 2 sets of 15 inspiratory efforts in order to minimize the time spent during warm-up and improve its application in a sports/physical training environment^32,38,39^. However, there is a lack of knowledge about the effects of different inspiratory loads of IMW on the mechanical and physiological performance in high-intensity and short-duration running, especially when performing such a short inspiratory protocol.

Therefore, this study aims to investigate the effects of different IMW loads (15%, 40% and 60% of MIP) using a shorter protocol (2 sets of 15 repetitions with a 1-min rest interval between them) on the mechanical (i.e., power, force and velocity), physiological (i.e., heart rate and blood lactate) and muscle oxygenation responses (in vastus lateralis and biceps brachii, considered the more and less active muscles, respectively) during high-intensity running and passive recovery. Our hypothesis is that IMW at 40% of MIP will minimize IM fatigue, allowing redirect the oxygenation to more active muscles with higher percentages of tissue saturation index (TSI), consequently promoting a positive impact on the mechanical responses during the 30 s all-out run (especially on mean running power and fatigue index), as well as contributing to a better post-effort blood lactate removal in relation to other loads. In addition, as studies are controversial and there are gaps about the IMW effects on high-intensity running, compared with the responses already observed in the literature in different populations and types of exercise it is estimated that the load with 15% of MIP has a placebo effect and is equal to the control session, while the 60% load does not improve performance, as it is considered a heavy load that will fatigue the respiratory muscles.

## Materials and methods

### Ethics approval

All procedures were approved by the local Research Ethics Committee of the School of Medical Sciences of the State University of Campinas (protocol number: 99783318.4.0000.5404) and were in accordance with the ethical recommendations in the Declaration of Helsinki. Participants were only evaluated after having received information about the experimental procedures and risks and signing an informed consent form.

### Participants

Sixteen physically active young men (local sports team players and street running participants) were evaluated (23 ± 1 years old, 73.2 ± 2.0 kg; 177.4 ± 1.9 cm; 9.0 ± 0.5% body fat, IPAQ at 4539 ± 942 metabolic equivalent-min/week; MIP at 145.6 ± 9.9 cmH_2_O, peak of global strength index (S-Index) at 139 ± 3 and mean S-Index at 123 ± 4, cmH_2_O). The analysis with G*Power software showed that a sample size of at least 12 individuals would be necessary to obtain a power of 80% with a significance level α = 5%, based on previously published data^10^. The participants were invited to answer questionnaires about their levels of physical activity (International Physical Activity Questionnaire—IPAQ), sports practice and health history. Only participants that presented a minimum score to classify them as ‘physically active’ were included in the study^42^. Individuals that reported metabolic, cardiovascular, respiratory or orthopedic disease were excluded from this research.

### Experimental approach to the problem

**Figure 1:**
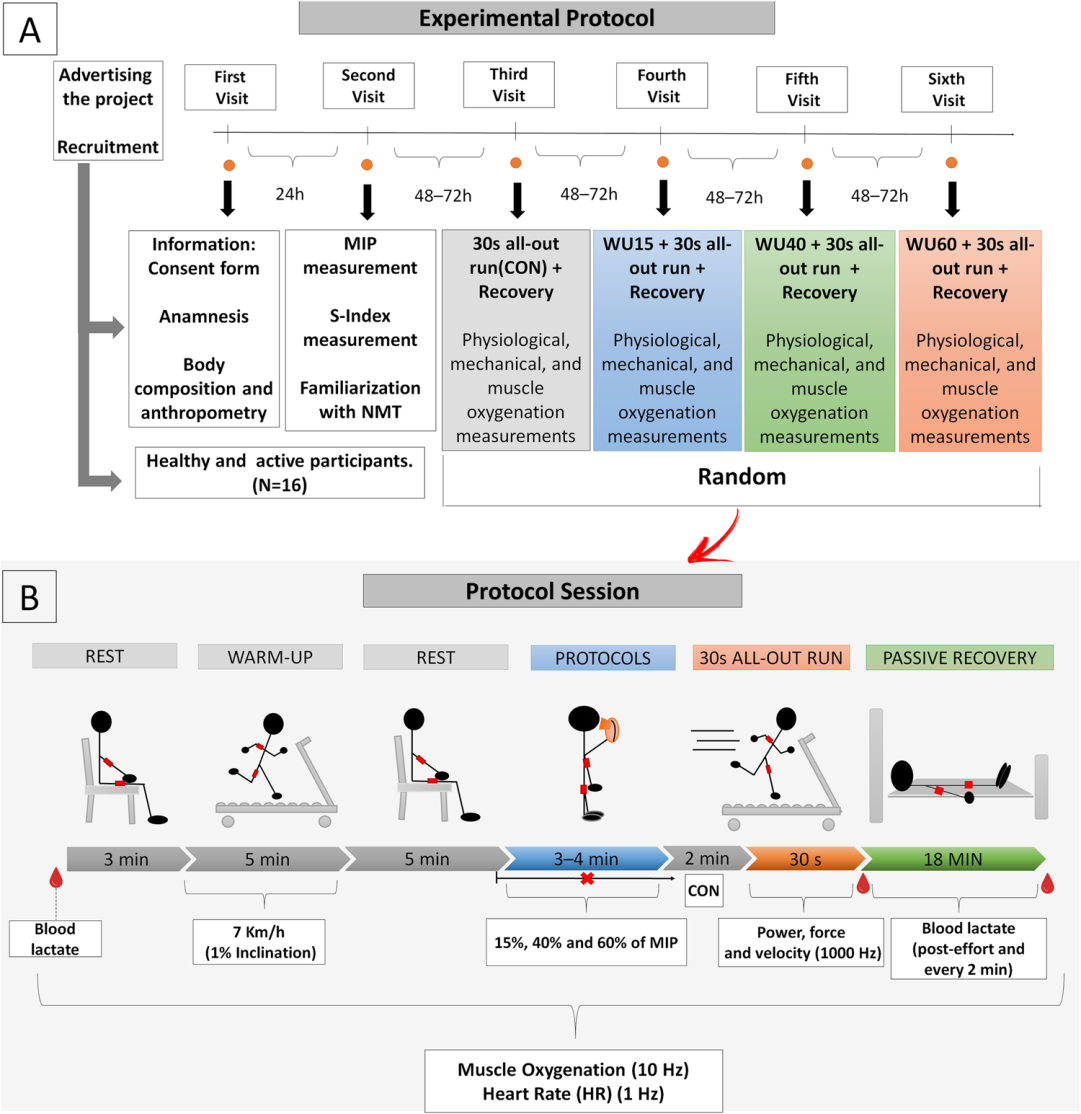
(**A**) Experimental protocol illustrating the procedures performed on each day during the whole study period. (**B**) Timeline of the protocol sessions. Physiological responses were monitored throughout the exercise and the 18 min of passive recovery. Near-infrared spectroscopy devices (NIRS, represented by the red rectangles) provided the biceps brachii and vastus lateralis oxygenation data. S-Index—global strength index; CON—Control session; WU15, WU40 and WU60—Warm-up with 15%, 40% and 60% of maximal inspiratory pressure (MIP), respectively.

The experimental design involved six visits to the laboratory under similar conditions and at identical daytime (± 60 min), separated by 48–72 h (Fig. 1). On the first day, after signing the informed consent form and completing the questionnaires, anthropometric and body composition measurements were performed. In the second visit, MIP and S-Index were determined 1 h apart to prevent inspiratory fatigue. On the same day, the participants were familiarized with the inspiratory muscle warm-up protocol and the non-motorized treadmill (NMT), when they were asked to perform five sprints of 10 s. In the next four visits to the laboratory, all participants were submitted to high-intensity tethered exercise (30 s all-out run), preceded by different IMW loads protocol (15%, 40% and 60% of MIP plus a control (CON) session without the IMW protocol). These sessions were randomly performed by the individuals under the IMW protocols used. Upon arrival at the laboratory, the participants were equipped with near-infrared spectroscopy (NIRS) devices attached to the biceps brachii (BB) and vastus lateralis (VL) muscles, and a heart rate monitor (HR) for data acquisition throughout the session. The participants remained at rest for 3 min for the determination of baseline values (BL), including blood lactate concentration ([Lac]) at rest. Then, they were asked to warm-up on a motorized treadmill for 5 min (7 km/h and 1% inclination) and rest for another 5 min. Subsequently, the IMW protocol was performed. Two minutes after, the individuals were submitted to 30 s high-intensity tethered running for data acquisition (i.e., force, velocity and running power). Immediately after the test (T0), blood samples were collected every 2 min up to 18 min of passive recovery (T2‒T18). In all sessions, the participants were instructed to have a light meal, not to consume alcohol/caffeine and not to practice moderate-intense exercise 24 h before the tests. The procedures were performed in a controlled and isolated laboratory environment, and the participants did not receive any information about each intervention.

### Inspiratory measurements and inspiratory muscle warm-up

The analysis was conducted by a trained researcher who demonstrated the correct performance of the respiratory maneuver. The participants remained seated on a chair, wearing a nose clip and a plastic mouthpiece connected to an analogical manovacuometer (± 300 cmH_2_O; GER-AR, São Paulo, SP, Brazil) used to measure maximal pressures. A small hole (2 mm) was introduced in the rigid mouthpiece in order to prevent glottic closure. The participants were instructed to complete three to five acceptable and reproducible maximum maneuvers (i.e., differences of 10% or less between values), with 1 min interval between maneuvers^43,44^. Each inspiratory effort was sustained for at least 1 s, and the MIP was considered the highest value between these attempts^45^.

After 1 h, a dynamic proposal to characterize the strength of the participants’ IM, the global strength index (S-Index), was assessed by an inspiratory threshold (POWERbreathe K5, IMT Technologies Ltd., Birmingham, UK), with the participants in standing position and using a nose clip. Thirty dynamic inspirations resistance-free were performed slowly, with verbal encouragement to inhale a greater air capacity^32^. During the protocol, breathing pattern curves were monitored by graphic records provided by *Breathe-link*® software. At the end of the test, the algorithm provided the mean and peak values of the S-Index (in units of cmH_2_O).

The inspiratory muscle warm-up (IMW) protocol loads were also applied using the inspiratory device POWERbreathe K5. The participants initiated every breath from residual volume and were encouraged to continue the respiratory effort until further excursion of the thorax was not possible, with a diaphragmatic breathing pattern. Subsequently, they were instructed to keep the same inspiratory pressure and the breathing pattern curves were also monitored by *Breathe-link*® software. The total protocol was comprised of two sets of 15 inspirations with a 1-min rest interval between them. The loads were equivalent to 15% (WU15), considered placebo by the literature^32,35,39^, 40% (WU40) and 60% (WU60) of MIP. All experimental trials were randomly distributed.

### The 30 s all-out run test and mechanical measurements

The 30 s all-out run was performed on a non-motorized treadmill (NMT) (Inbramed Super ATL, Inbrasport, Porto Alegre, Brazil), as detailed by Manchado-Gobatto et al.^10^. Two minutes after the IMW or CON protocols, the participants were asked to run at maximum intensity for 30 s, tethered by their waist to an inextensible steel cable attached to a load cell (CSL/ZL-250, MK Controle e Instrumentação Ltda, Brazil) for horizontal force measurement. Other four load cells (CSAL/ZL-500, MK Controle e Instrumentação Ltda, Brazil) were positioned under the NMT platform to measure the vertical force (signal frequency at 1000 Hz). A hall-effect sensor in the frontal axis of the NMT provided pulses for velocity acquisition. Therefore, both vertical and horizontal force components were measured during the running exercise along with velocity to calculate the power running. The signals were synchronized and the product between force and velocity resulted in the running power, with the peak, mean and minimum values relativized to body mass. Fatigue index (FI) was also calculated by the following equation: FI = (peak power—minimum power)/peak power * 100). The system was calibrated on the test days.

### Physiological responses

#### Blood lactate concentration and heart rate

For lactate concentrations ([Lac]) at rest, post-effort and every 2 min up to 18 min of passive recovery, blood samples (25 µl) were collected from the participants’ earlobe with heparinized capillaries, deposited in microtubes (Eppendorf, 1.5 ml containing 50 µl of 1% sodium fluoride—NaF) and frozen at − 20 °C. The [Lac] were determined by a lactate analyzer (YSI-2300-STAT-Plus™, Yellow Springs, USA). Throughout the protocols, the heart rate (HR) was constantly recorded (at 1 Hz) (Polar V800, Finland). For all variables, the peak, mean and minimum values were calculated (during the test we used the 30 s responses, while during passive recovery we considered the mean of 18 min).

### Muscle oxygenation by NIRS measurements

Total hemoglobin ([tHb] = oxyhemoglobin ([O_2_Hb]) + deoxyhemoglobin [HHb]) and the equilibrium between oxygen supply and consumption were calculated using the tissue saturation index (TSI = [O_2_Hb]/([O_2_Hb] + [HHb]) × 100%)^46^ throughout the experimental protocol by two PortaMon devices (Artinis, Medical Systems BV, Zetten, Netherlands) working on the modified Beer–Lambert law. Each device has three light source transmitters (with two wavelengths of 760 and 850 nm), positioned at 30, 35 and 40 mm from the receiver. The devices were safely fixed and covered to eliminate background light after shaving and cleaning the skin surface. While one was positioned in the medial part (belly) of the BB^10,37,47^ of the right arm, considered less active during running, the other was allocated in the VL of the right leg (considered more active), 15 cm above the proximal edge of the patella and 5 cm to the external side^10,48–50^. Skinfolds for BB (3.3 ± 0.2 mm) and VL (11.2 ± 1.2 mm) were less than half the distance between the source and the deepest detector (i.e., 20 mm). Different path lengths (DPF) were used for BB (3.78) and VL (3.83)^10^. The signals were smoothed using a 10^th^ order low-pass zero-phase Butterworth filter (cutoff frequency of 0.1 Hz)^50^**,** and recorded and analyzed on Oxysoft® software (Artinis Medical System, Netherlands). The (Δ)[tHb] in micromolar units (μM) was obtained by subtracting these values from the final 30 s of the baseline period of 3 min. Examples of NIRS signals in BB and VL muscles of one participant during the four sessions (CON, WU15, WU40 and WU60) are displayed in Supplementary file 1, whereas the descriptive graphics of [O_2_Hb] and [HHb] during and after the 30 s all-out run are in Supplementary file 2.

### Statistical analysis

All analyses were performed using Statistica software (version 7.0), and the results are expressed as mean ± standard error of the mean (SEM). Data normality and homogeneity distribution were tested by Shapiro–Wilk and Levene’s test, respectively. Two-way repeated measures analysis of variance (ANOVA) was applied to study the effect of IMW (CON x WU15 x WU40 x WU60) and time during the 30 s of exercise (on mechanical and physiological responses) as well as the 18 min (every 2 min) of passive recovery (on physiological responses) compared to the baseline condition (BL). Three-way repeated measures ANOVA investigated the effect of IMW, time, and limb muscles (BB *vs* VL) on muscle oxygenation during the 30 s all-out run and recovery phase. One-way repeated measures ANOVA was applied to investigate the effects of IMW protocols on peak, mean, minimum values for force (N/kg), velocity (m/s), running power (W/kg), FI, as well HR and [Lac] values. Two-way repeated measures ANOVA (effects of IMW and limb muscles) analyzed the peak, mean and minimum values of muscle oxygenation responses. Lastly, post hoc Newman−Keuls test was used to detect differences (P ≤ 0.05). The partial eta-squared (η_p_^2^: 0.01 = small, 0.06 = moderate, 0.14 = large) statistics provided estimates of the effect sizes.

## Results

### Analyses during the 30 s all-out run

**Figure 2:**
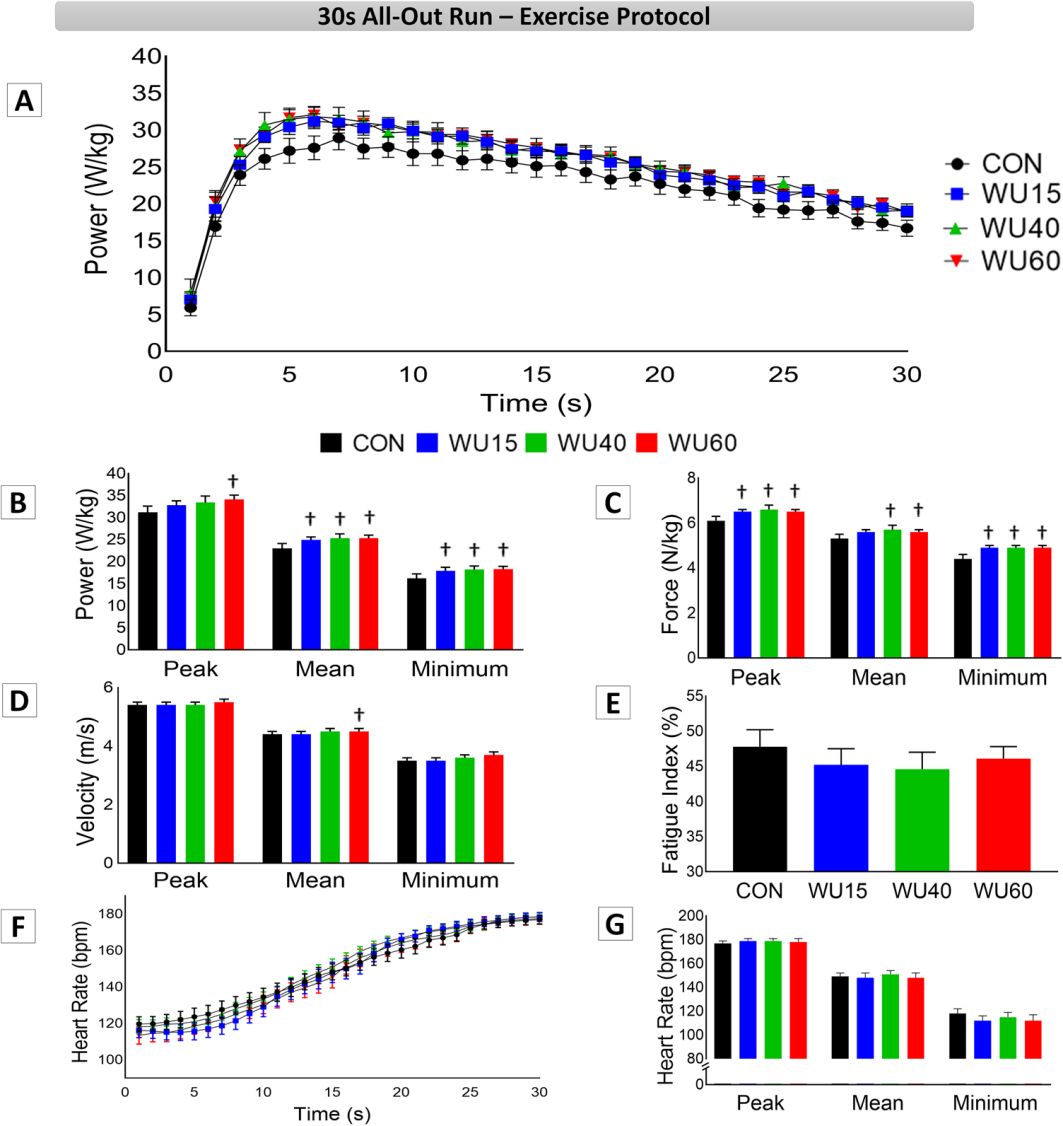
Mechanical and physiological results observed during the 30 s all-out run under control conditions (CON, black color) and after IMW loads at 15% (WU15, blue color), 40% (WU40, green color) and 60% of MIP (WU60, red color). **✝** Indicates difference in relation to the control session. (P < 0.05).

The mechanical and heart rate results during the 30 s all-out run are shown in Fig. 2. Panel A shows similar running power responses between interventions during the 30 s all-out run test. The two-way repeated measures ANOVA demonstrated a significant effect of time on this parameter (F_(29,1740)_ = 255.50, P ˂ 0.001, η_p_^2^ = 0.790). There was an increase in running power in the first second until a peak power was reached at approximately 6 s and a consequent decrease after this time for all interventions, without any effects of IMW on the studied parameter. Panels B, C, D and E display the peak, mean and minimum values for power, force, velocity and FI, respectively. For these measurements, the one-way repeated measures ANOVA revealed an effect of IMW (F_(3,45)_ = 2.98, P = 0.041, η_p_^2^ = 0.166) on peak power (Panel B), with higher values in WU60 (34.1 ± 1.0 W/kg, P = 0.029) compared to CON (31.2 ± 1.4 W/kg). For mean power (Panel B; F_(3,45)_ = 4.5, P = 0.007, η_p_^2^ = 0.232), minimum power (Panel B; F_(3,45)_ = 3.3, P = 0.028, η_p_^2^ = 0.181), peak force (Panel C; F_(3,45)_ = 3.9, P = 0.013, η_p_^2^ = 0.208), minimum force (Panel C; F_(3,45)_ = 5.5, P = 0.002, η_p_^2^ = 0.269), all IMW loads led to higher values in comparison with CON. Additionally, regarding specific IMW effects on mean force (Panel C; F_(3,45)_ = 3.2, P = 0.028, η_p_^2^ = 0.180), higher values were observed in WU40 (5,6 ± 0.1 N/kg, P = 0.035) and WU60 (5.6 ± 0.1 N/kg, P = 0.020) than in CON (5.2 ± 0.1 N/kg), whereas a higher mean velocity value (Panel D; F_(3,45)_ = 3.5, P = 0.022, η_p_^2^ = 0.190) was obtained for WU60 (4.5 ± 0.1 m/s) than for CON (4.3 ± 0.1 m/s). Panel F represents the HR responses second by second during the 30 s all-out run. The two-way repeated measures ANOVA pointed to a significant effect of time (F_(29,1740)_ = 350.76, P ˂ 0.001, η_p_^2^ = 0.854), yet no effects among interventions were detected. The HR constantly increased, reaching the highest values in the last second of the test. Furthermore, the peak, mean and minimum HR values were not influenced by IMW (Panel G).

**Figure 3:**
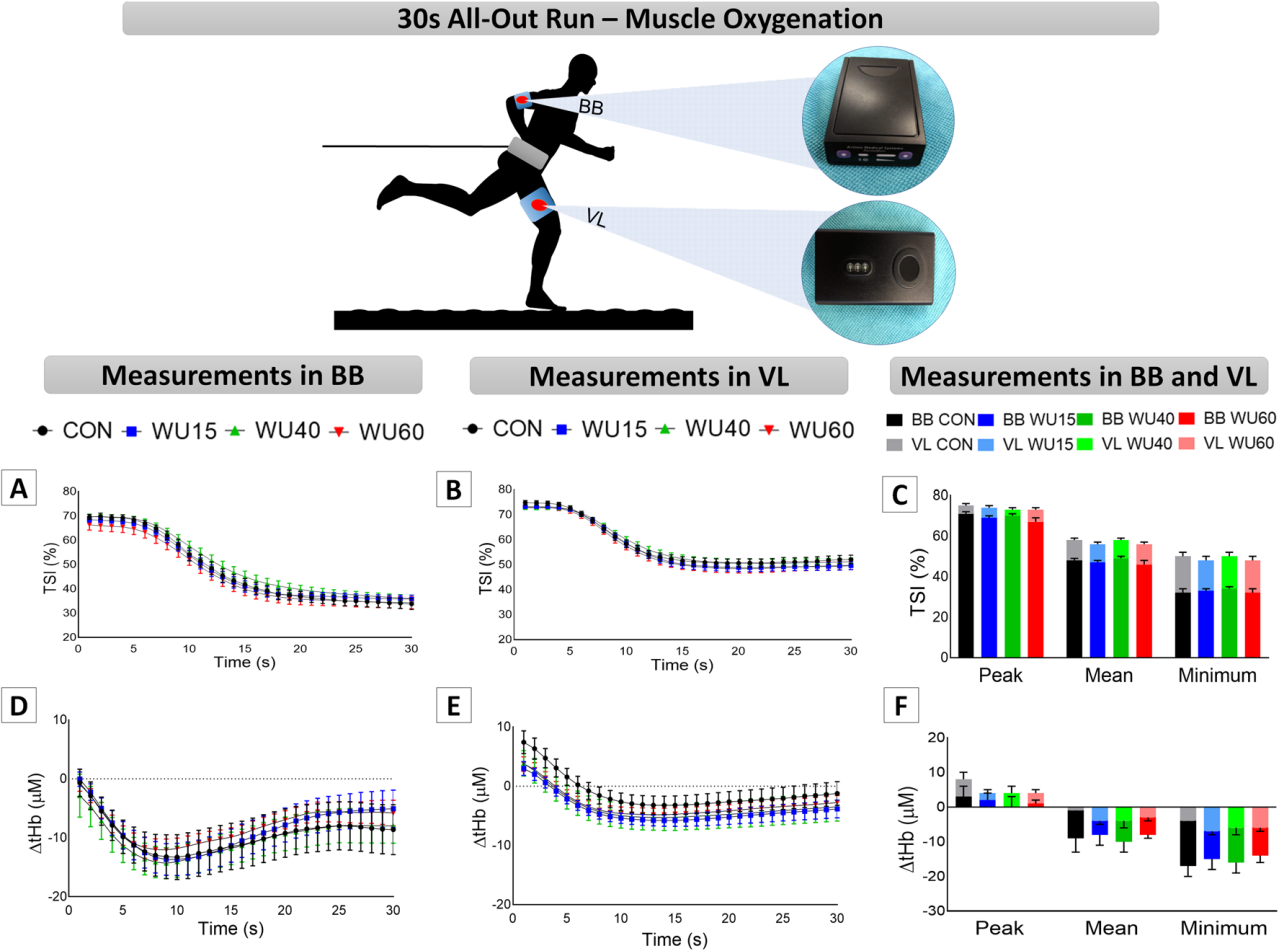
Results of tissue saturation indexes (TSI, Panels **A–C**) and total hemoglobin ([tHb], Panels **D–F**) in biceps brachii (BB) and vastus lateralis (VL) at each second during the 30 s all-out run on a non-motorized treadmill under control conditions (CON, black color) and after the IMW loads with 15% (WU15, blue color), 40% (WU40, green color) and 60% of MIP (WU60, red color), in line graphs. In bar graphs, the dark colors represent the BB, while the light colors correspond to the VL. MIP = maximum inspiratory pressure.

The changes in muscle oxygenation are shown in Fig. 3. Panels A and B illustrate the TSI responses in BB and VL, respectively. The three-way repeated measures ANOVA revealed effects of limb muscle (F_(1,120)_ = 85.83, P ˂ 0.001, η_p_^2^ = 0.419), time (F_(29,3480)_ = 928.57, P ˂ 0.001, η_p_^2^ = 0.886) and significant interaction between time x limb muscle (F_(29,3480)_ = 36.39, P ˂ 0.001, η_p_^2^ = 0.233), but no IMW effects. The peak, mean and minimum TSI data (Panel C) were analyzed by two-way ANOVA, which indicated the effect of limb muscle (TSI; peak: F_(1,30)_ = 20.67, P ˂ 0.001, η_p_^2^ = 0.408; mean: F_(1,30)_ = 33.49, P ˂ 0.001, η_p_^2^ = 0.527 and minimum: F_(1,30)_ = 71.53, P ˂ 0.001, η_p_^2^ = 0.705) and IMW only for TSI mean values (F_(1,90)_ = 3.72, P = 0.014, η_p_^2^ = 0.110). Despite these findings, no interaction between IMW and limb muscles was observed.

The Δ [tHb] for BB and VL are presented in Panels D and E. The three-way repeated measures ANOVA showed the effects of limb muscle (F_(1,119)_ = 15.03, P ˂ 0.001, η_p_^2^ = 0.112), time (F_(29,3451)_ = 82.66, P ˂ 0.001, η_p_^2^ = 0.410), and an interaction between time x limb muscle (F_(29,3451)_ = 16.86, P ˂ 0.001, η_p_^2^ = 0.124). However, no difference was observed through post-hoc analyses (IMW x time x muscle limb). Whereas for peak values no differences were observed, for mean (F_(1,30)_ = 10.00, P = 0.003, η_p_^2^ = 0.250) and minimum values (Panel F; F_(1,30)_ = 23.73, P ˂ 0.001, η_p_^2^ = 0.442) a limb effect was detected without interaction with IMW.

### Analyses in passive recovery

**Figure 4:**
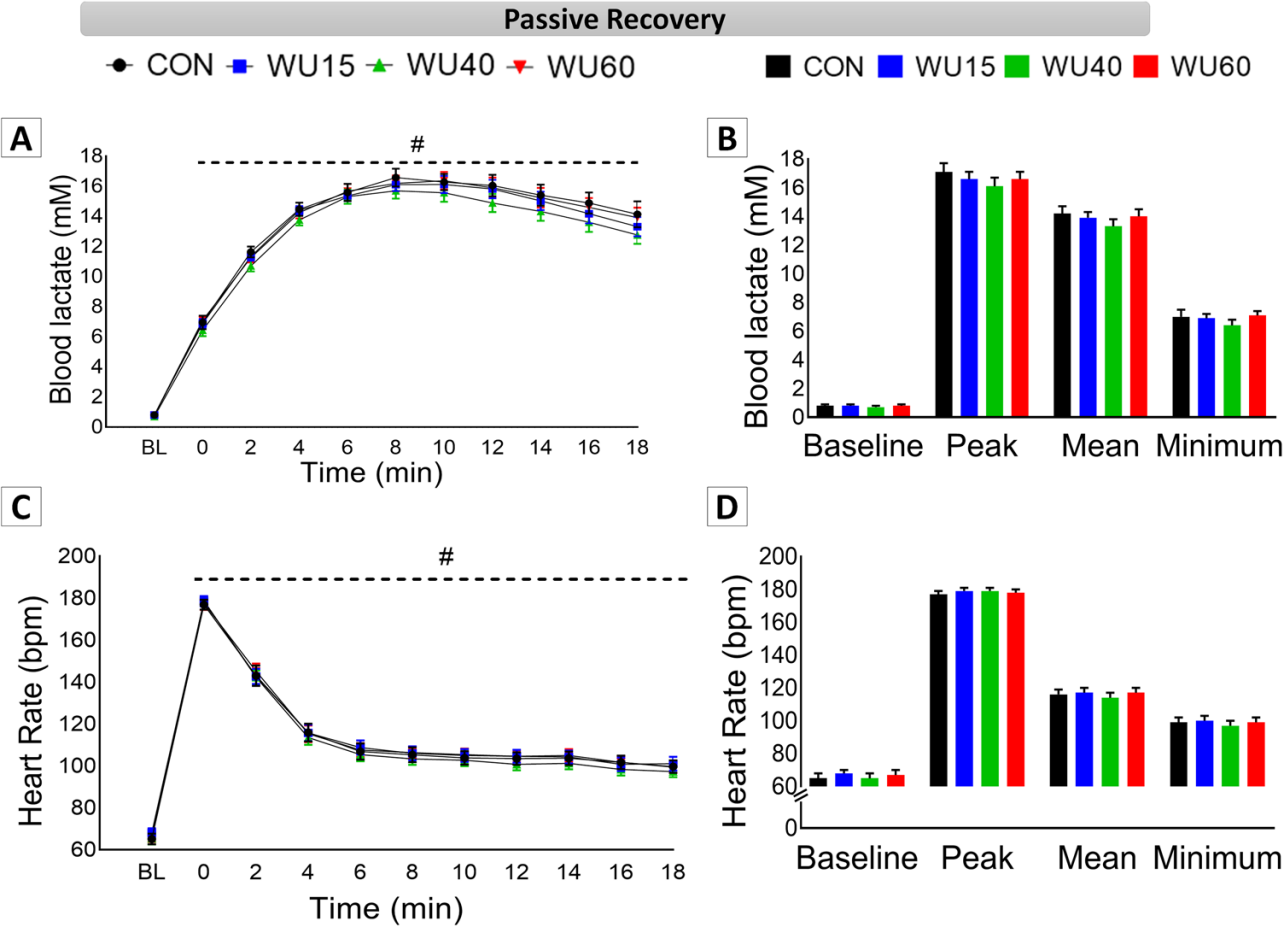
Physiological results of passive recovery immediately after the 30 s all-out run and every 2 min up to 18 min (mean ± EPM). Results under control conditions (CON, black color) and after IMW protocols with loads at 15% (WU15, blue color), 40% (WU40, green color) and 60% of MIP (WU60, red color). **#** corresponds to difference in relation to baseline (BL) for each protocol. Dashed line means no differences among protocols.

Physiological responses obtained at baseline (BL) immediately after the 30 s all-out run (T0) and during passive recovery (every 2 min: T2–T18) are displayed in Fig. 4. Panel A shows that the curves of blood lactate were similar in all interventions, with a clear effect of time (F_(10,600)_ = 1053.76, P ˂ 0.001, η_p_^2^ = 0.946). A [Lac] peak was observed at 8 min for all participants ([Lac] peak in CON: 17.14 ± 0.62; WU15: 16.00 ± 0.51; WU40: 16.11 ± 0.56 and WU60: 16.60 ± 0.65, mM). No effects of IMW were observed. [Lac] values did not return to their initial values (BL) after 18 min of recovery, independently of the IMW load applied. A similar HR behavior was observed for all conditions (Panel C), with a significant effect of time (F_(10,600)_ = 1646.51, P ˂ 0.001, η_p_^2^ = 0.965). The HR peak was obtained immediately after the 30 s all-out run (post-effort, T0), reaching higher values than T2–T18 and decreasing over time. After 18 min, the HR values did not reach the initial values under any condition. Peak, mean and minimum values for [Lac] and HR are shown in panels B and D, respectively. IMW was not able to influence these results.

**Figure 5:**
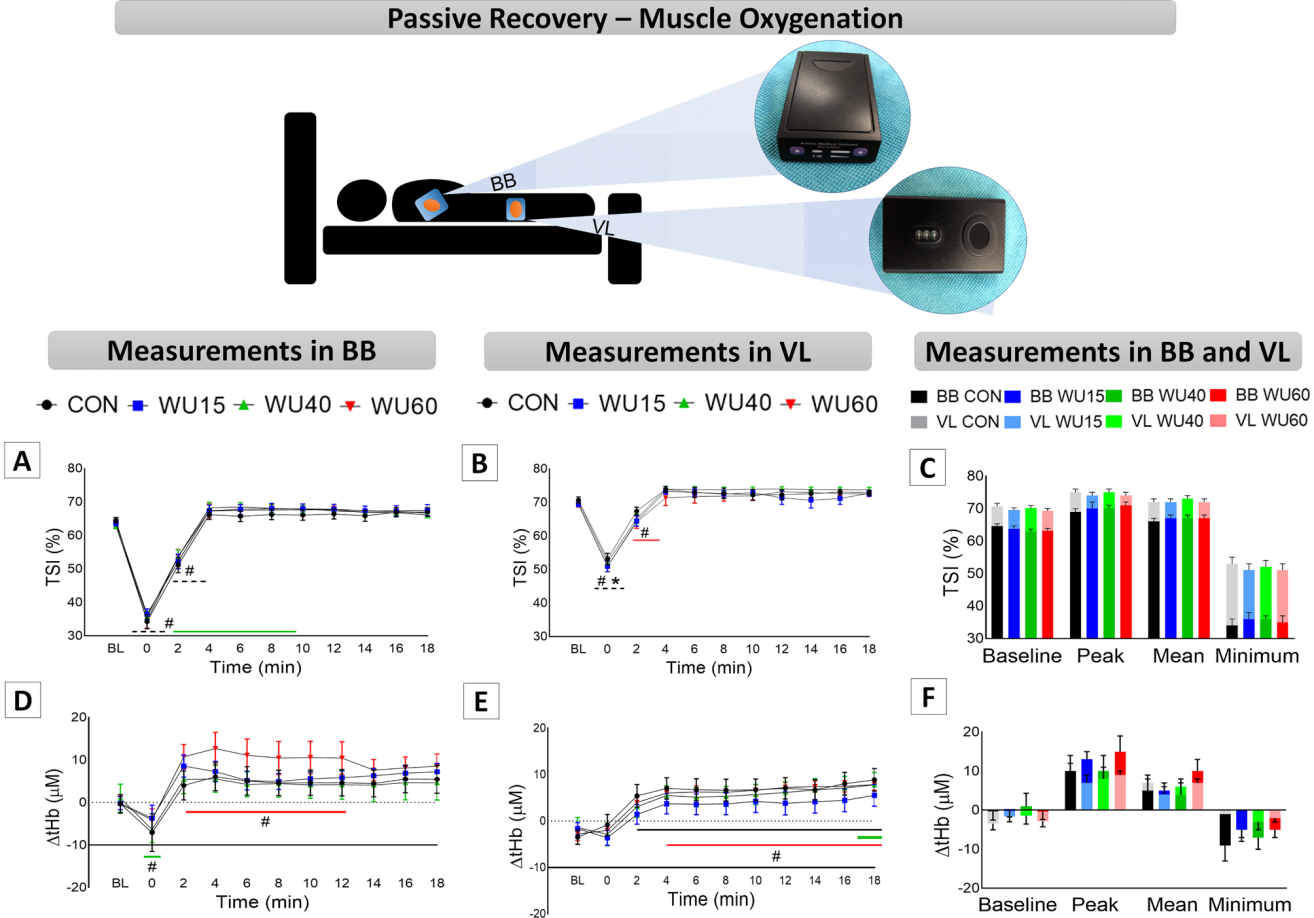
Results of tissue saturation indexes (TSI, Panels **A–C**) and total hemoglobin ([tHb], Panels **D–F**) in passive recovery immediately after the 30 s all-out run (T0) and every 2 min up to 18 min (T2–T18) compared to BL values. Timeline panels show the biceps brachii (BB) and vastus lateralis (VL) under control conditions (CON, black color) and after the IMW loads with 15% (WU15, blue color), 40% (WU40, green color) and 60% of MIP (WU60, red color). Bar graphs present the peak, mean and minimum values (mean ± SEM), with dark colors for BB and light colors for VL. MIP = maximum inspiratory pressure. **#** corresponds to difference compared to baseline (BL); * means significantly different from BB. Dashed line means no differences among protocols. Colored line means differences in a specific protocol.

Changes in muscle oxygenation during passive recovery are shown in Fig. 5. Panels A and B display the TSI responses in BB and VL, respectively. The three-way repeated measures ANOVA revealed effects of limb muscle (F_(1,120)_ = 93.23, P ˂ 0.001, η_p_^2^ = 0.437), time (F_(10,1200)_ = 643.46, P ˂ 0.001, η_p_^2^ = 0.843) and a significant interaction between time x limb muscle (F_(10,1200)_ = 32.43, P ˂ 0.001, η_p_^2^ = 0.213), but no interaction with IMW. In all interventions, BB presented lower saturation in relation to BL at T0 and T2, and after 4 min of recovery (T4) the saturation returned to BL values. An interesting finding was observed in WU40, which reached higher oxygenation values from T4 to T10 compared to BL. In VL, only WU60 at T2 presented different values from BL. Moreover, higher saturation was detected in VL than in BB at T0. The two-way repeated measures ANOVA revealed an effect of limb muscle on BL (F_(1,30)_ = 61.16, P ˂ 0.001, η_p_^2^ = 0.671), peak (F_(1,30)_ = 17.87, P ˂ 0.001, η_p_^2^ = 0.373), mean (F_(1,30)_ = 30.06, P ˂ 0.001, η_p_^2^ = 0.501) and minimum (F_(1,30)_ = 55.17, P ˂ 0.001, η_p_^2^ = 0.648) values (Panel C) without IWM interaction.

Regarding [tHb] (Panel D‒E), there were a significant effect of time (F_(10,1200)_ = 44.73, P ˂ 0.001, η_p_^2^ = 0.272) and interaction between time x limb muscle (F_(10,1200)_ = 2.95, P ˂ 0.001, η_p_^2^ = 0.024). Compared to BL values, the passive recovery time showed lower values for BB at T0 only in WU40, while higher values were noted at T2–T12 in WU60, subsequently returning to BL values. In VL, differences from BL values were observed at specific moments in CON (T2‒T8), WU40 (T18) and WU60 (T4‒T18). IMW strategies did not affect peak, mean and minimum [tHb] values in passive recovery (Panel F).

## Discussion

To the best of our knowledge, this is the first study dedicated to investigating the effects of different IMW loads (15, 40 and 60% of MIP) on mechanical and physiological responses, including oxygenation in more and less active muscles, during and after high-intensity, short-duration running exercise. Additionally, we studied these acute inspiratory strategies using a shorter protocol (i.e., lower number of exercise repetitions). Our main findings revealed some effects of IMW, performed with 2 sets of 15 repetitions with a 1-min rest interval between the sets, on the high-intensity running effort and recovery, independently of the load applied. Regarding the mechanical parameters, all IMW promoted a significant increase in mean and minimum running power, as well as in peak and minimum running force compared to CON. Additionally, when applying specific IMW loads higher values were observed for peak power, mean velocity (WU60) and mean force (WU40 and WU60) in relation to CON. The physiological responses, including HR and oxygenation in more and less active muscles during the running exercise, were not modified by IMW, at least not during the 30 s high-intensity running nor for HR and [Lac] in the post-effort phase. By comparing the responses in BB and VL, no differences were observed in mechanical and muscle oxygenation during the 30 s all-out test. In passive recovery, higher TSI values for VL were detected in the post-effort phase (T0) for all protocols. An interesting finding was observed in WU40, which reached higher oxygenation values from T4 to T10 compared to BL. It can be then suggested that the use of different loads of IMW promotes an improvement in performance corroborated by the increased peak, mean and minimum mechanical values, but not in the performance assessed second by second. Also, WU40 may improve recovery phase with higher oxygenation in BB.

### Inspiratory muscle warm-up and performance

Our choice to investigate the effect of different IMW loads on the performance of high-intensity, short-duration running exercise was based on previous studies that indicate the positive effect of IMW, but used different IWM protocols in intermittent running^27^, in Wingate tests^33,34^, in 100 m freestyle swimming^29^, in specific hockey drills^31^ and in a simulate judo match^32^. Additionally, considering the large use of tethered efforts in physical and sports programs together with the significance of high-intensity exercise in this context, we focused on the evaluation of the IMW impact on the force, velocity and running power of 30 s all-out run sessions using different inspiratory loads. As shown in Fig. 2, the same characteristics were observed for the curve of running power throughout the tests, with no differences among the IMW load interventions during the 30 s all-out run sessions. As previously mentioned, running power, force and velocity were improved by the IMW loads, more specifically the WU40 and WU60, which significantly influenced the mechanical variables compared to CON, suggesting an improvement in running performance for active participants. Regarding the exercise performance of athletes, the IMW combined with specific warm-up was capable of reducing the time in 100 m freestyle swimming^29^, treadmill sprint performance^51^ and interactions among the technical-tactical, physical, physiological, and psychophysiological parameters in a simulated judo match^32^. Studies that used IMW as the only means of prior effort to main motor task also observed a reduction in the sensation of dyspnea and an improvement in the distance walked in one badminton-footwork test^36^, as well as in one shuttle run test^52^. Similarly, Özdal and colleagues^34^ observed an increase in peak and relative power in anaerobic Wingate test performed by hockey athletes after IMW (2 × 30 at 40% of MIP with a 2-min rest interval between the sets).

Some additional positive effects of IMW on respiratory response and performance in a shuttle run test^27^, on respiratory muscle strength^28,53^, on performance in a knee flexion–extension protocol accomplished in an isokinetic test by healthy sedentary participants^41^ and on long-distance test^30^ were previously observed. On the other hand, other studies did not find significant effects of IMW for both active individuals and athletes performing different types of exercise and tests^39,43,46,53–56^. It is important to consider the diversity of the IMW protocols when applied to different populations, sports modalities and exercise tests, which makes it difficult to compare the results obtained with other findings. Moreover, most investigations do not describe the time interval between the IMW sets and the time between the IMW application and the test or main exercise. Knowing that the effects of warm-up can be affected by several factors, such as protocol, load, performance level, type of exercise, time interval between the conditioning stimulus and the performance testing, etc.^57,58^, more attention could be paid to these aspects. In this sense, we focused on a shorter IMW protocol (2 sets of 15 repetitions with a 1-min rest interval between them, concluded 2 min before the running test), using different inspiratory loads in each session (without and with 15, 40 and 60% of MIP) applied to active participants. Regarding the respiratory parameters, even though our participants did not have any experience with respiratory training or inspiratory warm-up, they reached good MIP values (145.6 ± 9.9 cmH_2_O), similar to Japanese athletes in triathlon and wrestling (light category) modalities (145.8 and 147.3 cmH_2_O, respectively)^59^.

### Inspiratory muscle warm-up and physiological responses

The ventilatory responses in high-intensity exercises can affect the perfusion dynamics of the locomotor muscles and tissue saturation indices, representing a limitation of exercise performance^19,20,60^. According to the literature, inspiratory muscle warm-up can be a strategy to potentialize oxygenation redistribution to more active muscles during physical exercise^35^. However, improvements in post-effort recovery process remain unexplored. Thus, we measured for the first time the physiological responses in exercise and recovery, including oxygenation analysis in more or less active muscles (which are relevant to providing oxygen and removing metabolites, respectively). Few scientific investigations, especially in sports, have been conducted to study the IMW potential to minimize respiratory fatigue and improve the oxygenation redistribution in high-intensity exercise^35,40,46,51,61^. When studying the oxygenation in the gastrocnemius muscle of female soccer players by submitting them to submaximal cycling test and intermittent cycling test, Cheng et al.^35^ demonstrated that the IMW protocol can enhance oxygen saturation in this tissue. However, the authors did not observe changes in performance, possibly due to the lack of specificity in the test for these athletes.

In our study, second-by-second NIRS analyses did not reveal the effects of IMW on muscle responses, regardless of the load (Fig. 3, Panels A‒B). Despite the evidence of limb muscle effect on TSI peak, mean and minimum values (Fig. 3, Panel C) and [tHb] mean and minimum values (Fig. 3, Panel F), no interaction with IMW protocols was observed, indicating a similar behavior in BB and VL oxygenation. Whether the IMW promotes a positive effect on the oxygenation redistribution^35^, this was not observed in more and less active muscles. Considering the increased respiratory muscle work and the competition with locomotor muscles for O_2_ supply^17–20^, the analysis of inspiratory muscle oxygenation could provide some insight into the oxygenation between these muscles. However, the analysis of oxygenation occurs only in accessory and secondary inspiratory muscles^62^, and it does not directly reflect the oxygenation of the muscle with the greatest potential for oxygen uptake and the most affected one by IMW, the diaphragm. Recently, studies addressing the IMW applied to speed skaters on ice time trial also did not report any improvement in muscle oxygenation variables in the right VL, with some limitations pointed out by the authors, such as leg compression garments and small sample size^40,46^. On the other hand, in high-intensity sprint^10^ and high-intensity cycling^37^ a difference in more and less active muscles was observed, suggesting adjustments in oxygenation during effort in a tissue-dependent manner.

In order to support the high demand of the respiratory muscles during exercise, the VO_2_ and oxygenation in this region are increased, and may compromise cardiac output by 14–16% in well-trained individuals^63^, thus affecting oxygenation distribution to locomotor muscles^64^. In high-intensity effort, these locomotor muscles also use predominantly anaerobic pathways, resulting in lactate production. According to previous studies, inspiratory muscles may play an important role as lactate consumers^21,22^. In this context, Lin et al.^36^ indicated a reduction in [Lac] in badminton players after IMW. Regarding [Lac], the peak values observed here (~ 16 mM) confirmed their significant anaerobic contribution in the 30 s all-out run. These findings corroborate previous studies on exercises characterized by anaerobic contribution^9,10,65,66^. In our study, both HR and [Lac] were not affected by IMW interventions, and 18 min post-effort in passive recovery was not sufficient to make these physiological responses return to baseline values (Fig. 4, Panels A–D).

With respect to passive recovery, a higher decrement in oxygenation was observed immediately after the exercise, the so-called post-effort phase (T0), with quick adjustments after T2 for both muscles (Fig. 5). Osawa et al.^37^ reported that tissue oxygenation did not begin immediately after high-intensity cycling effort and that deoxygenation occurred for a few seconds. In the present study, TSI percentages started to rise immediately after the exercise, and after 4 min (T4) they returned to baseline values (Fig. 5, Panels A‒B). Interestingly, only WU40 presented higher TSI values in BB from T4 to T10 in relation to BL. We did not perform correlation analyses, however, Manchado-Gobatto et al.^10^ observed a significant correlation between the [Lac] peak and TSI and [tHb] values in BB. In this regard, higher TSI in BB suggests an important role of this parameter during recovery since O_2_ is considered to be essential in oxidative pathways for lactate clearance^67^. In VL, only WU60 at T2 reached lower values than BL. Despite our initial hypothesis, which proposed that 40% of MIP would redirect oxygenation to VL with a consequent positive impact on mechanical responses during a tethered 30 s all-out run, in addition to a better blood lactate removal during recovery in relation to other loads, we observed that different inspiratory loads can improve mechanical parameters and recovery oxygenation.

Recent studies investigated the effects of IMW as a warm-up strategy combined with core warm-ups on recovery period between intermittent exercise and repeated sprints on NMT^51^ and on recovery periods of sprints on a cycle ergometer^68^. Although the authors evaluated recovery, muscle oxygenation was only observed during exercise. In our study, the comparison between BB and VL revealed that only immediately after the 30 s all-out run the VL presented higher values for all interventions (Fig. 5, Panels A–B). These results corroborate those reported by Manchado-Gobatto et al.^10^, who did not use inspiratory strategy to improve the running performance. Finally, the responses during exercise and recovery are a complex process. Thus, to improve the interpretation of IMW on running and recovery, integrative analyses could reveal responses beyond conventional statistics. For example, our group recently observed the improvement in the technical and tactical parameters in a judo simulated fight using the same shorter IMW protocol used herein, based on a complex network analysis^32^. In such study, the centrality metrics revealed that the IWM at 15% of MIP favored the interactions among the psychophysiological, physical and physiological parameters, while the IWM at 40% of MIP was able to improve performance in the judo match. Therefore, our next investigations will be considering these findings.

Furthermore, a recent study indicated NIRS measurements as a future physiological marker, showing no significant differences regarding the respiratory compensation point^69^. These findings highlight the relationship between systemic (i.e., ventilatory) and peripheral (i.e., oxygenation of locomotor and non-locomotor muscles) physiological breakpoints. In this sense, future studies should consider respiratory strategies associated with the NIRS technique to improve knowledge about the intensity of the training zone. Moreover, the IMW can attenuate muscle deoxygenation during exercise^35^ and the NIRS technique can contribute to the monitoring of oxygenation in clinical practice^70^, especially in patients with exercise-induced ischemic pain caused by reduced blood flow to the lower extremities^71^.

## Limitations and strengths

Despite the use of technologies with high-frequency signal acquisition, some limitations regarding our results must be addressed. First, the all-out run performed on a NMT has shown reliable results in the scientific literature^5,7–10,66,72^. However, we did not test the reproducibility of the four IMW interventions – although it is safe to say that they exhibited similar results in the 30 s all-out run tests (Fig. 2) with no differences in power running among the IMW loads second by second. We chose to investigate a classic anaerobic test (30 s all-out run), considering the aerobic component around ~ 18–20% in these efforts^7,13^. We observed an effect of IMW on the mechanical parameters, which did not result in greater muscle oxygenation differentiation. It is possible that by applying another slightly longer exercise protocol or repeated sprints the impact of IMW can be observed on both mechanical parameters (second by second) and physiological responses. Additionally, we did not use the gas analyzer to investigate the oxygen uptake due to our experimental design, nor investigated oxygenation of the inspiratory muscles. Still, we are aware that the association of NIRS measurements and VO_2_ exchange would improve our data interpretation, but we have not tested whether this equipment can interfere with breathing pattern or breath frequency altering the isolated effects of IMW. Another limitation refers to our participants' characteristics, as only healthy active males, non-athletes performed the test. In future studies, we suggest the inclusion of female participants, the comparison of IMW with a shorter protocol and the analysis of effects in both high-performance athletes and non-athletes in different types of exercises, such as repeated-sprint effort.

The strengths of this study include: (i) the investigation of the inspiratory muscle strategy through a shorter protocol applied with methodological rigor, performed with practical and high-quality inspiratory devices, (ii) the running effort performed on a non-motorized treadmill able to identify, with high signal capture, minimal changes in mechanical variables during high-intensity exercise; (iii) monitoring oxygenation responses in different muscle groups, associated or not with respiratory strategies, allows a more integrative interpretation of this variable during effort and also during recovery.

## Conclusion

In summary, different IMW loads with a shorter protocol (2 sets of 15 repetitions with a 1-min rest interval between sets and 2 min before exercise) applied on high-intensity running exercise suggested an improvement in performance corroborated by increased peak, mean and minimum mechanical values, but not in power and oxygenation assessed second by second. With respect to muscle oxygenation, these measurements demonstrated that the mechanisms by which IMW could possibly exert an effect on performance were not affected by these protocols, as all interventions showed similar and rapid adjustments of oxygenation responses during exercise demands. Interestingly, during passive recovery WU40 presented a pronounced TSI value for BB, indicating a greater availability of O_2_ for lactate clearance in a tissue-dependent manner.

## Supplementary Information

Supplementary Information.

## Data Availability

The data that support the findings of this study are available from the corresponding author on reasonable request. Correspondence and requests for materials should be addressed to F.B.M.G.
