# Supplementary material for: Effects of different inspiratory muscle warm-up loads on mechanical, physiological and muscle oxygenation responses during high-intensity running and recovery
Source: Sci Rep. 2022 Jul 2;12:11223. doi: 10.1038/s41598-022-14616-w (PMC9250525; doi:10.1038/s41598-022-14616-w)
Supplement: Supplementary file 1 — Supplementary Information. [file 41598_2022_14616_MOESM1_ESM.docx]

**
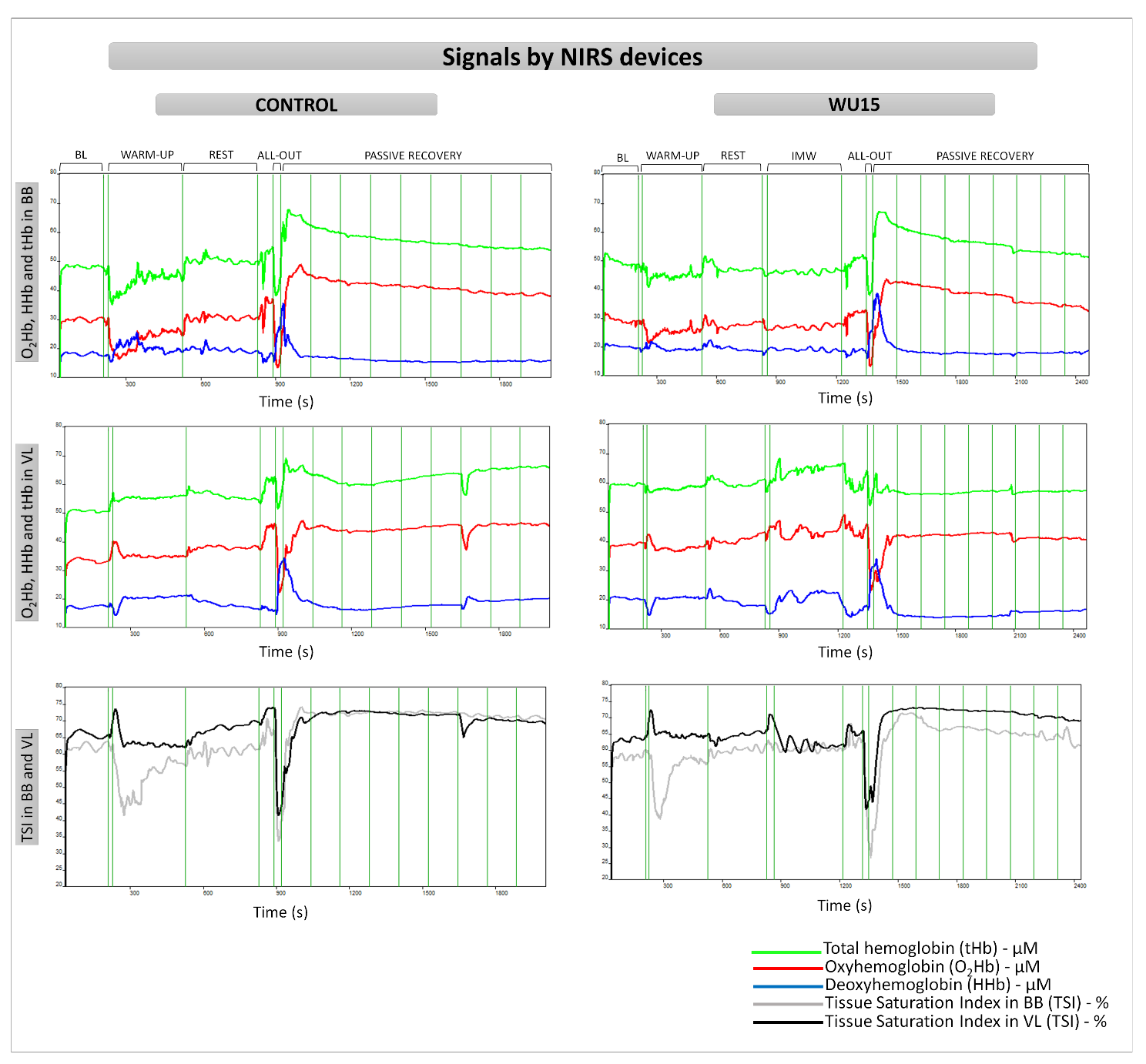
SUPPLEMENTARY FILE**

**Supplementary file 1 (part 1).** Data for illustrative purposes of an example of NIRS signal in the Biceps brachii (BB) and Vastus lateralis (VL) muscles of one participant submitted to the 30s all-out run without (control) and with 15% (WU15) of maximal inspiratory pressure. The keys delimit moments performed in the protocol session. The graphs were provided by Oxysoft® software (Artinis Medical System, Netherlands), with automatic scaling in absolute and percentage concentrations. These data were not relativized by the baseline values.


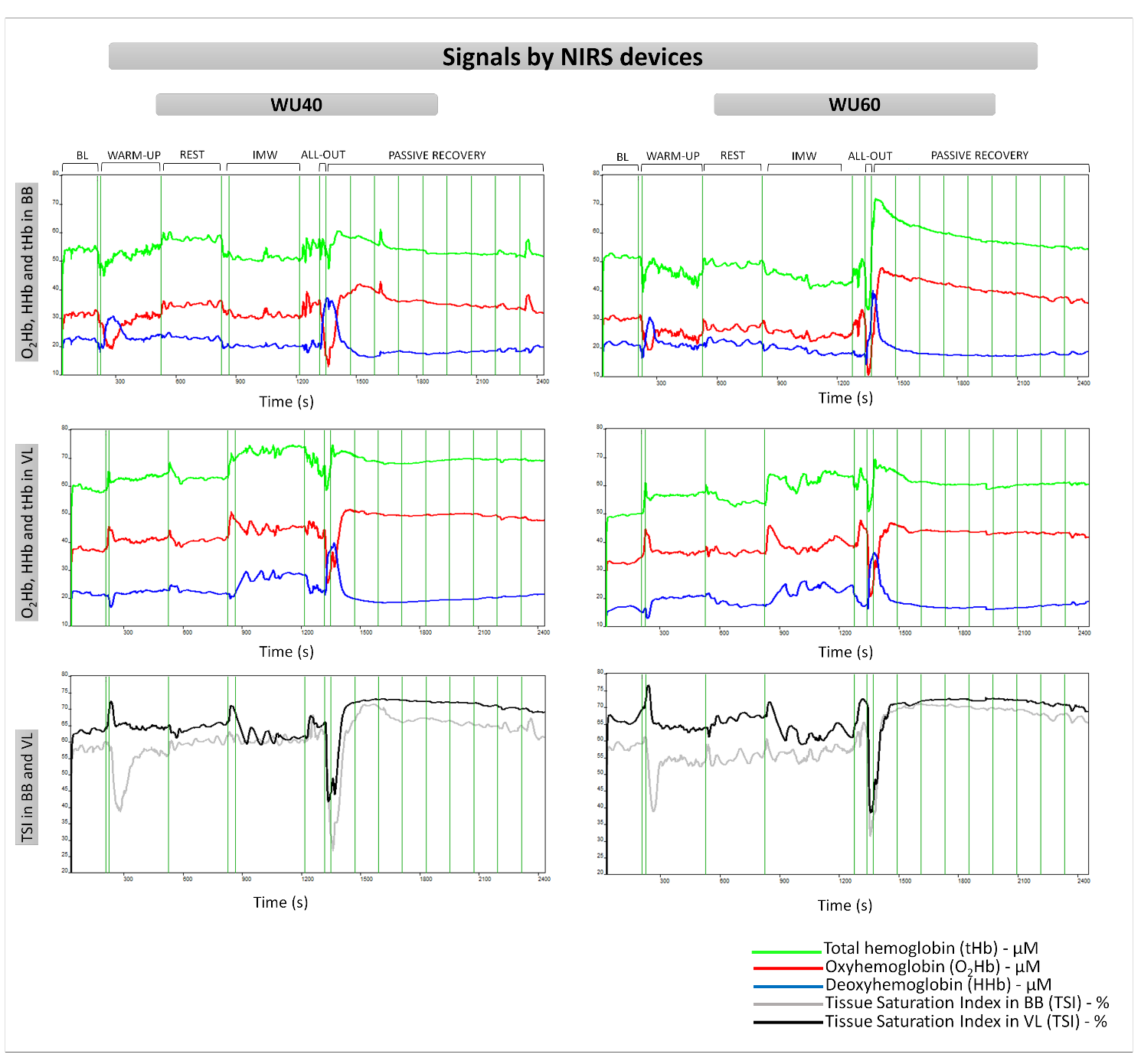


**Supplementary file 1 (part 2).** Data for illustrative purposes of an example of NIRS signal in the Biceps brachii (BB) and Vastus lateralis (VL) muscles of one participant submitted to the 30s all-out run with 40% (WU40) and 60% (WU60) of maximal inspiratory pressure. The keys delimit moments performed in the protocol session. The graphs were provided by Oxysoft® software (Artinis Medical System, Netherlands), with automatic scaling in absolute and percentage concentrations. These data were not relativized by the baseline values.


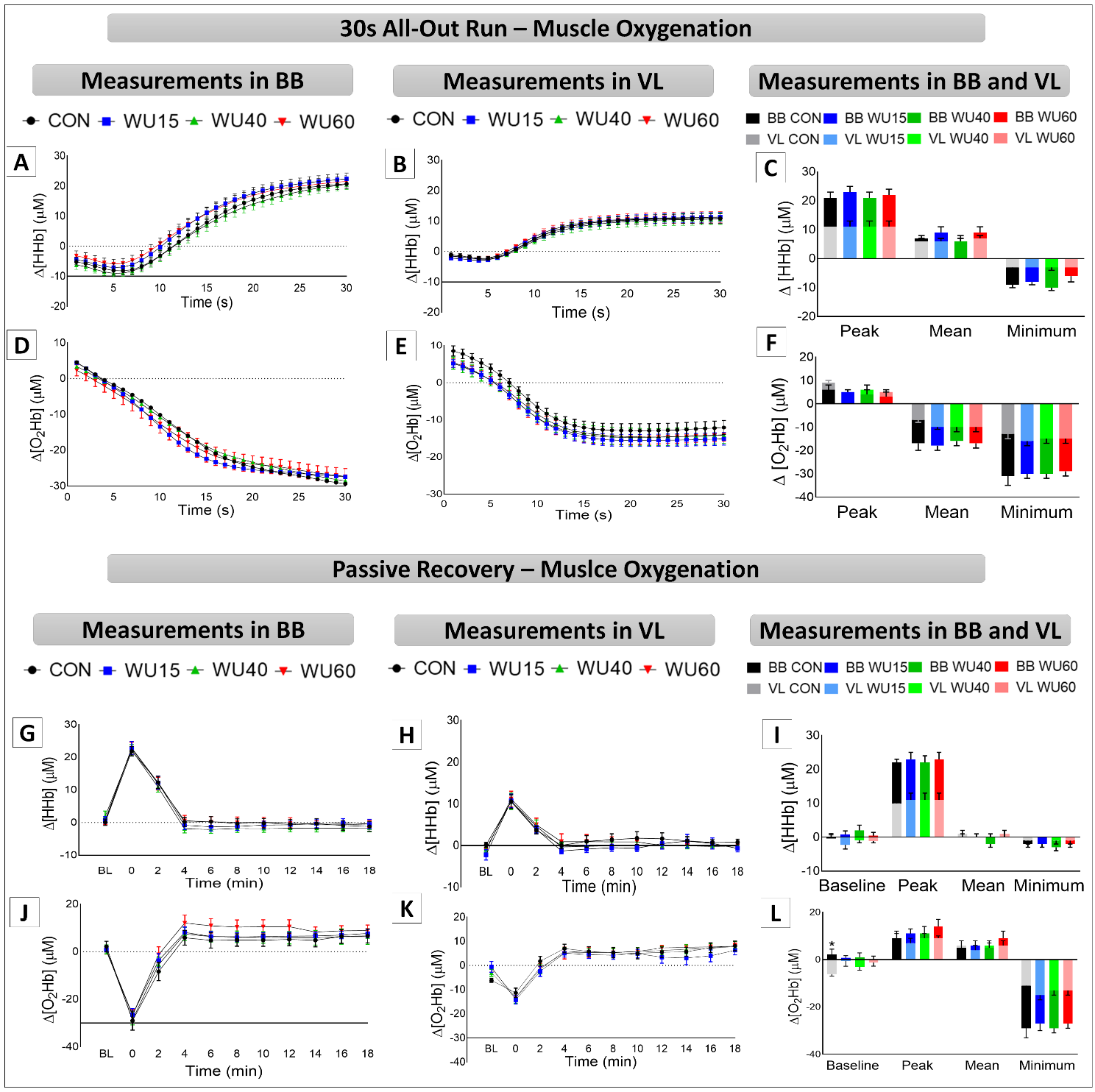


**Supplementary file 2.** Descriptive graphics illustrating the results of deoxyhemoglobin ([HHb]) and oxyhemoglobin ([O_2_Hb]) in 30s all-out run (Panels A–C and D–F, respectively) and passive recovery (Panels G–I and J–L, respectively) in Biceps brachii (BB) and Vastus lateralis (VL) under control conditions (black color) and after IMW loads with 15% (blue color), 40% (green color) and 60% of MIP (red color). Bar graphs present the peak, mean and minimum values (mean ± SEM), with dark colors for BB and light colors for VL. MIP = maximum inspiratory pressure.
